# Traditional Chinese Medicine Formulation Huangqi Jianzhong Tang Improves Cardiac Function after Myocardial Infarction in Rats

**DOI:** 10.1155/2020/3106076

**Published:** 2020-10-09

**Authors:** Ya-Ru Bao, Wen-Yi Jiang, Jia-Yu Yu, Jing-Wei Chen, Guo-Xing Zhang

**Affiliations:** ^1^Department of Physiology, Medical College of Soochow University, 199 Ren-Ai Road, Dushu Lake Campus, Suzhou Industrial Park, Suzhou 215123, China; ^2^Department of Internal Medicine, The Affiliated Suzhou Chinese Traditional Medicine Hospital, Nanjing University of Chinese Medicine, 18 Yang-Su Road, Suzhou 215003, China

## Abstract

Huangqi Jianzhong Tang (HQJZT) is a traditional Chinese herbal formula consisting of seven different herbs: Radix Astragali, Radix Paeoniae Alba, Ramulus Cinnamomi, Fructus Jujubae, Glycyrrhizae Radix Et Rhizoma Praeparata Cum Melle, Rhizoma Zingiberis Recens, and Saccharum Granorum. The present study aims to evaluate the possible effects of HQJZT on cardiac function in acute myocardial infarction (AMI) and related mechanism. AMI model was established by ligation of the left anterior descending coronary artery followed by one-week HQJZT treatment. Survival rate was calculated. Rat heart function was assessed by heart performance analysis system. 5-Triphenyltetrazolium chloride (TTC) staining was used to observe myocardial infarct size. Terminal deoxynucleotidyl transferase-mediated dUTP-biotin nick end labeling (TUNEL) staining and western blot were applied to evaluate tissue apoptotic level. Treatment with high dose of HQJZT improved cardiac function, reduced infarct size, number of apoptotic cells and expression of apoptotic proteins, Bax (a proapoptotic protein), and increased expression of antiapoptotic protein, Bcl2. However, enalapril (an angiotensin-converting enzyme inhibitor) treatment did not show marked improvement of these parameters. Our present data suggest that HQJZT has potential therapeutic effects to improve cardiac function by regulation of apoptotic signaling pathway.

## 1. Introduction

Heart failure is the terminal stage of cardiovascular disease. Most of the myocardial infarction (MI) cases will finally develop into heart failure. MI has become one of the main causes of death in patients with cardiovascular diseases. The common consequence of MI is cardiac tissue death induced by heart dysfunction [1, 2]. As soon as possible to recover from cardiac tissue death becomes a critical issue. Nowadays, primary percutaneous coronary intervention (PPCI) has been developed to restore blood to rescue MI patients; however, reperfusion paradoxically induces myocardial injury and cardiomyocyte death, thereby mitigating the full benefits of PPCI [3]. Conservative treatment includes the application of antithrombotic drugs, beta-blockers, lipid-lowering drugs, nitrates, calcium antagonists, angiotensin-converting enzyme inhibitors (ACEI), and angiotensin II receptor-1 blockers; the clinical outcome of these treatments is still disappointed [4, 5]. Therefore, exploring novel additional or alternative therapies to satisfy clinical demands is still essentially necessary.

TCM has long history of more than 2000 years and has gained widespread clinical applications, including in treatment of cardiovascular disorders [6, 7]. Although TCM is considered to be complementary or alternative medicine in most of Western countries, it is increasingly welcomed in many developed countries, such as Australia [8] and the United States [9] due to its reputed effectiveness, low cost, and relative absence of side effects.

Huangqi Jianzhong Tang (HQJZT) is a traditional Chinese herbal formula consisting of seven different herbs: *Radix Astragali*, *Radix Paeoniae Alba*, *Ramulus Cinnamomi*, *Fructus Jujubae*, *Glycyrrhizae Radix Et Rhizoma Praeparata Cum Melle*, *Rhizoma Zingiberis Recens*, and *Saccharum Granorum*. It has been recorded on a classic TCM masterpiece “Jin Gui Yao Lue” (*Synopsis of Prescriptions of the Golden Chamber*), which was written by a TCM master, Zhang Zhong-Jing (Eastern Han Dynasty, A.D. 150–219). HQJZT is mainly used for the treatment of chronic gastrointestinal diseases such as chronic gastritis, peptic ulcers, inﬂammatory bowel disease, autonomic dystonia, chronic hepatitis, and chronic nephritis [10]. Basic research and clinical evidence suggest that HQJZT is a multitarget-directed agent based on its immunomodulatory, anti-inﬂammatory, and antioxidative effects [11–14]. Since dysregulation of immune signaling pathways, impaired suppression of postinfarction inflammation, and increase of oxidative stress contribute to the pathogenesis of heart failure after MI [15], it is suggested that HQJZT may help MI patients recover from heart dysfunction. In addition, modified HQJZT prescriptions, such as Shu-Mai-Tang, have been demonstrated to attenuate TNF*α*-induced myocardial fibrosis in myocardial ischemia rats [16]. Dang Gui Bu Xue Tang showed potent cardioprotective effects in rat heart mitochondria and erythrocytes [17]; Shu-mai-tang and Dan-Chuan-Hong also have beneficial effects on angiogenesis, arteriogenesis, antiapoptosis, and improvement of cardiac function in rats with myocardial ischemia [16, 18]. However, there is still lack of clinical evidence and definitive mechanisms of action to demonstrate the role of HQJZT in cardiovascular diseases. In the present study, we treated acute MI (AMI) rats with different doses of HQJZT or enalapril (an angiotensin-converting enzyme inhibitor, ACEI) for one week. Cardiac performance and apoptotic signaling pathway were examined to assess the effect of HQJZT and to explore the related mechanism.

## 2. Materials and Methods

### 2.1. Preparation of HQJZT

HQJZT was composed of seven dried raw herbs (Table 1) prepared at Suzhou Chinese Traditional Medicine Hospital (Suzhou, China) and authenticated by a pharmacist of traditional Chinese medicine in Suzhou Traditional Chinese Medicine Hospital. All voucher specimens are deposited in the herbarium center of Suzhou Traditional Chinese Medicine Hospital. All raw materials were extracted by boiling in distilled water (about 6-fold weight of the mixture) at 100°C for 20 min and then filtered. The filtrates were stored at −80°C for further application. According to the General Guidelines of WHO for Methodologies on Research and Evaluation of Traditional Medicine, a sufficient number of dose levels should be used in rodents to determine the approximate lethal dose [19]. Two doses of HQJZT were applied in the present study: a low dose of 1.44 g/kg and a high dose 5.76 g/kg.

### 2.2. Experimental Animals

Ten-week-old male Sprague-Dawley rats were purchased from Shanghai Laboratory Animal Center (Shanghai, China). Rats were housed in optimal conditions with standard hygiene, kept at a temperature of 25°C with a 12/12 light/dark cycle, fed with standard rat chow and water ad libitum. The experiments were performed in accordance with the National Institutes of Health Guidelines for the Use of Laboratory Animals (NIH, publication number 85-23, revised 1996), which were in accordance with the guidelines for the care and use of animals established by Soochow University of Animal Care and Use Committee (Ethical Protocol no. 201912A75). Sodium pentobarbital anesthesia was used in all surgeries, and all efforts were made to minimize animal suffering.

### 2.3. Myocardial Infarction Model

The model was performed as in our previous report [20]. Briefly, rats were anesthetized with sodium pentobarbital (50 mg/kg, i.p.). Acute myocardial infarction (AMI) was performed by exposing the heart at the fifth intercostal space followed by a slipknot (6-0 silk) below the left descending coronary artery. Successful ligation was confirmed by ST segment elevation in postoperative ECG, compared with preoperative ones. After AMI surgery and waking from anesthetization, rats were gastrically treated without (*n* = 30) or with HQJZT solution for 1.44 g/kg/day (low dose, *n* = 30) or 5.76 g/kg/day (high dose, *n* = 30), or treated with Enalapril (0.18 mg/kg/day, *n* = 30, provided by Jiangyin Day Jiang pharmaceutical Co., Ltd., Suzhou, China). Dose of Enalapril applied in the present observation was based on previous observation, which was demonstrated to improve cardiac function in chronic left ventricular dysfunction rat model [21]. The doses of HQJZT were equal to 1-, 4-fold doses for clinical patient application calculated according to body surface area. Sham rats were without occlusion of left descending coronary artery (*n* = 10). Treatment period lasted for one week. Rats have then undergone cardiac function examination; after that, rats were sacrificed, and hearts were harvested for further analysis. Finally, each group included 10 rats.

### 2.4. Measurement of Cardiac Function

One week later, rats were anesthetized with sodium pentobarbital (50 mg/kg, i.p.); hemodynamic parameters were measured with a heart performance analysis system (ALCBIO, Shanghai Alcott Biotech Co., Ltd., Shanghai, China). Left femoral artery and right common carotid artery were isolated. A polystyrene PE-50 catheter (Bunzl Healthcare Co., Ltd., London, UK) was inserted into the left ventricle via right common carotid artery, and the other end was connected to the measurement system. The major parameters of cardiac function were derived or calculated from the continuously obtained pressure signal, including systolic arterial pressure (SAP), the rate of maximum positive and negative left ventricular pressure development (LV ± d*p*/d*t* max), the left ventricular end-diastolic pressure (LVEDP), etc.

### 2.5. Measurement of Infarction Size of Myocardial Tissue

After cardiac function was measured under anesthetized condition with sodium pentobarbital (50 mg/kg, i.p.), rats were sacrificed, and their hearts were excised immediately and perfused with Evans blue (1%, 4 ml) via the coronary artery. Hearts were transversely cut into 1-2 mm slices along the ligation point, placed in 1.25% 2,3,5-triphenyltetrazolium chloride (TTC; Sigma, USA) solution in PBS, incubated for 10 min at 37°C. The infarct tissue appeared white (not stained by TTC) and the normal tissue was red (stained by TTC); the samples were recorded by using a digital light microscope (Olympus, Tokyo, Japan). The ratio of myocardial infarct area (white part) to whole section area (left and right ventricle section) was calculated using NIH image software (Rawak Software, Inc., Stuttgart, Germany).

### 2.6. Terminal Deoxynucleotidyl Transferase-Mediated dUTP-Biotin Nick End Labeling Assay (TUNEL) Staining

Rat hearts were sampled and fixed with 3.7% paraformaldehyde in PBS, embedded in paraffin, and cut into 5 *μ*m slices. Apoptotic cells were detected by using the terminal deoxynucleotidyl transferase-mediated dUTP nick end labeling (TUNEL) assay kit (Calbiochem, Darmstadt, Germany) according to the manufacturer's instructions. Total number of 1,000 cells was counted; the mean percentage of TUNEL-positive cell was calculated.

### 2.7. Western Blot Analysis

Myocardial tissues were homogenized with radioimmunoprecipitation assay (RIPA) buffer (50 mm Tris, ph 7.0, 150 mM NaCl, 1% Triton-X-100) containing phenylmethanesulfonyl fluoride (R&D Systems Inc., Minneapolis, USA). Homogenates were centrifuged at 12,000 × *g* for 10 min at 4°C. Target proteins were separated by SDS-PAGE and transferred to polyvinylidene fluoride (PVDF) membranes (Hybond™-ECL; Amersham Pharmacia Biotech, Inc., Shanghai, China). The membranes were blocked in 5% nonfat milk in PBS and 0.1% Tween-20 at room temperature. The blots were then incubated with primary antibody: anti-Bcl-2 antibody (1:1000, ImmunoWay Biotech, Inc., Shanghai, China), anti-Bax antibody (1:1000, Abcam, Inc., Shanghai, China), anti-Caspase-3 antibody (1:1000, Abcam, Inc., Shanghai, China), or anti-GAPDH (Santa Cruz Biotech, Inc., Shanghai, China) for 2 h at room temperature or at 4°C overnight. Membranes were then washed for 15 minutes using TBS-T, three times. Washing was done to remove excess antibody before incubation for 1 minute with chemiluminescent reagents (ECL, R&D Systems Inc., Minneapolis, USA). Membranes were subsequently exposed to X-ray film. Immunoreactive bands were detected by analysis of X-ray films using the software Image J (Rawak Software, Inc., Stuttgart, Germany). The quantity of target proteins was normalized to GAPDH expression.

### 2.8. Statistical Analysis

The SPSS 18.0 software (IBM, Inc., Shanghai, China) was used for statistical analysis. Data are presented as the mean ± standard error mean (SEM). Grouped data was analyzed using a two-way or one-way analysis of variance followed by the Student–Newman–Keuls test for each group. A *P* value <0.05 was considered statistically significant.

## 3. Results

### 3.1. Effect of HQJZT on Mortality

**Figure 1:**
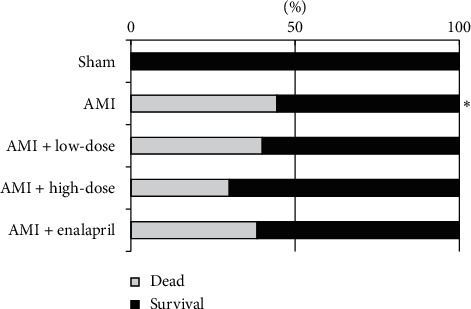
Statistical analysis of mortality in each group. Percentage of dead rats in each group was calculated after one week. Sham represents without ligation of the left descending coronary artery group (*n* = 10), AMI represents acute myocardial infraction group (*n* = 30), AMI + low dose represents acute myocardial infarction treated with low dose of HQJZT group (*n* = 30), AMI + high dose represents acute myocardial infarction treated with high dose of HQJZT group (*n* = 30), and AMI + enalapril represents acute myocardial infarction treated with enalapril group (*n* = 30). ^*∗*^*P* < 0.05 compared with sham group.

Mortality during one-week observation period was analyzed and the statistical data showed that mortality in AMI group was significantly higher compared with sham group (*P* < 0.05). There was no significant difference among all groups subjected to AMI (*P* > 0.05, Figure 1).

### 3.2. Effect of HQJZT on Cardiac Function

To assess the effect of HQJZT on cardiac function in rats subjected to AMI, we performed cardiac function measurements one week after ligation. AMI significantly decreased cardiac function compared with the sham group, by decreasing the diastolic arterial pressure (DAP) and mean arterial pressure (MAP), *P*_max_, ± d*p*/d*t*_max_ (Table 2). One-week treatment with low dose of HQJZT only improved −d*p*/d*t*_max_ when compared with AMI group. High-dose treatment with HQJZT improved DAP and ±d*p*/d*t*_max_ when compared with AMI group. Enalaprilat treatment showed the similar effect with low dose of HQJZT on cardiac function parameters, only improving −d*p*/d*t*_max_.

HR: heart rate; RRI: the R-R interval; SAP: systolic arterial pressure; DAP: diastolic arterial pressure; MAP: mean arterial pressure; PP: pulse pressure; *P*_max_: the maximum of left ventricular pressure development; *P*_min_: the minimum of left ventricular pressure development; *P*_mean_: the mean of ventricular pressure development; LVEDP: left ventricular end-diastolic pressure; +d*p*/d*t*_max_: rates of maximum positive left ventricular pressure development; and −d*p*/d*t*_max_: rates of maximum negative left ventricular pressure development. Sham represents without ligation of the left descending coronary artery group (*n* = 10), AMI represents acute myocardial infraction group (*n* = 10), AMI + low dose represents acute myocardial infarction treated with low dose of HQJZT group (*n* = 10), AMI + high dose represents acute myocardial infarction treated with high dose of HQJZT group (*n* = 10), and AMI + enalapril represents acute myocardial infarction treated with enalapril group (*n* = 10). ^*∗*^*P* < 0.05 compared with sham group. ^#^*P* < 0.05 compare with AMI group.

### 3.3. Effect of HQJZT on Myocardial Infarct Size

**Figure 2:**
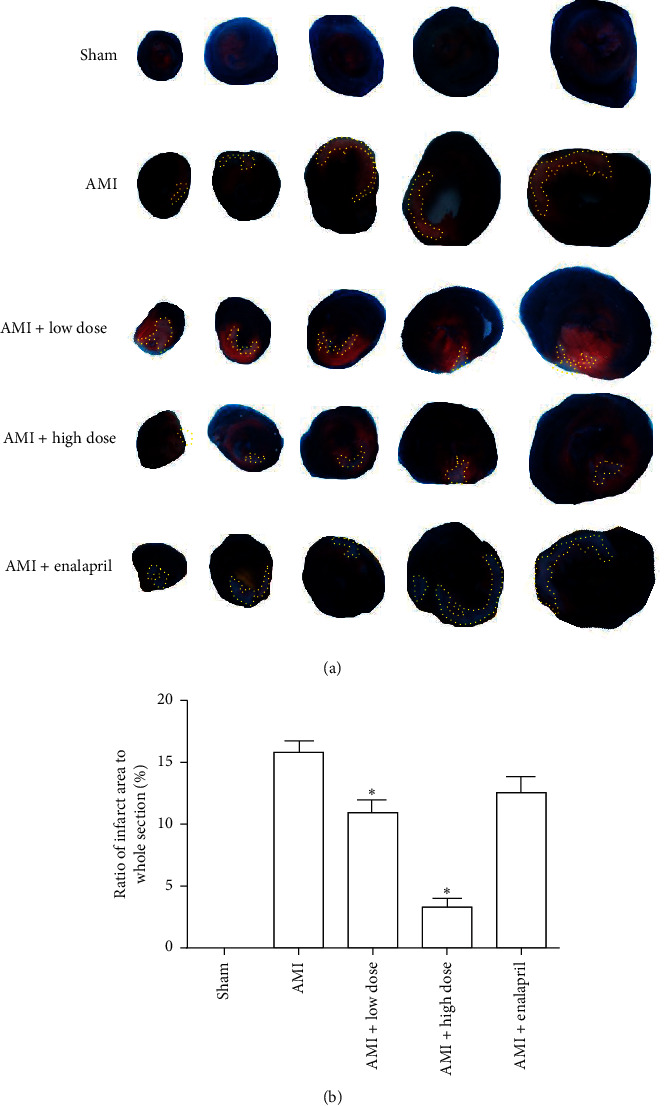
Effects of HQJZT on the infarct size. (a) Representative photo of heart cross sections in each group; area circled by yellow line indicates infarction area. (b) Percentage of infarcted area to whole section in each group. Sham represents without ligation of the left descending coronary artery group (*n* = 6), AMI represents acute myocardial infraction group (*n* = 6), AMI + low dose represents acute myocardial infarction treated with low dose of HQJZT group (*n* = 6), AMI + high dose represents acute myocardial infarction treated with high dose of HQJZT group (*n* = 6), and AMI + enalapril represents acute myocardial infarction treated with enalapril group (*n* = 6). ^*∗*^*P* < 0.05 compared with AMI group.

Cardiomyocyte injury is characterized by myocardial infarct size. To determine whether HQJZT attenuates cardiomyocyte injury, the ratio of infarct size to whole section was calculated. AMI group showed that the ratio of infarct size to whole section was 15.79 ± 0.92%. Treatment with low-dose HQJZT significantly reduced the ratio to 11.72 ± 1.82%; treatment with high-dose HQJZT further decreased the ratio to 3.30 ± 0.59% (Figure 2). However, treatment with enalapril tended to reduce the ratio; statistical analysis showed that there was no significant difference compared with AMI group (12.55 ± 2.62%, *P*=0.088). These results suggest that HQJZT ameliorates cardiomyocyte injury and improves cardiac recovery. In addition, HQJZT showed better effect than enalapril during early phase of cardiac infarction injury.

### 3.4. Effect of HQJZT on Apoptotic Cell Number

**Figure 3:**
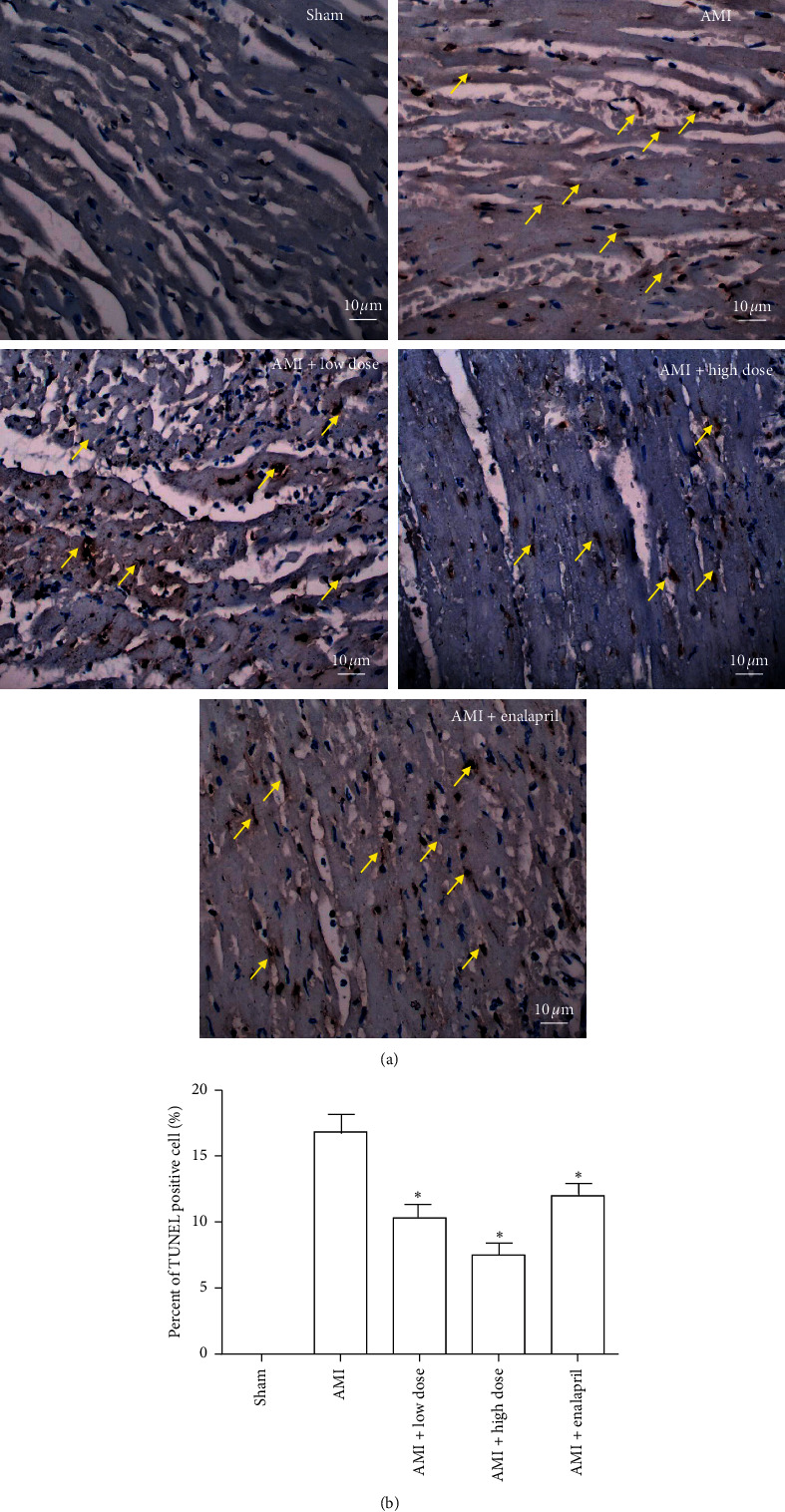
TUNEL staining in each group. (a) Representative image of TUNEL staining in heart tissue (magnification 400x). Yellow arrow indicates TUNEL-positive cell (brown staining). (b) Percent of TUNEL-positive cell in each group. Sham represents without ligation of the left descending coronary artery group (*n* = 4), AMI represents acute myocardial infraction group (*n* = 4), AMI + low dose represents acute myocardial infarction treated with low dose of HQJZT group (*n* = 4), AMI + high dose represents acute myocardial infarction treated with high dose of HQJZT group (*n* = 4), and AMI + enalapril represents acute myocardial infarction treated with enalapril group (*n* = 4). ^*∗*^*P* < 0.05 compared with AMI group.

TUNEL staining results showed that there were still large amounts of apoptotic cells after one-week AMI. Treatment with low-dose or high-dose HQJZT significantly reduced the percentage of TUNEL-positive cell. Enalapril treatment also reduced the percentage of TUNEL-positive cell (Figure 3). These results suggest that HQJZT accelerates cell repairment after AMI. ACEI also could improve cell repairment after AMI.

### 3.5. Effect of HQJZT on Apoptotic Signaling Pathway

**Figure 4:**
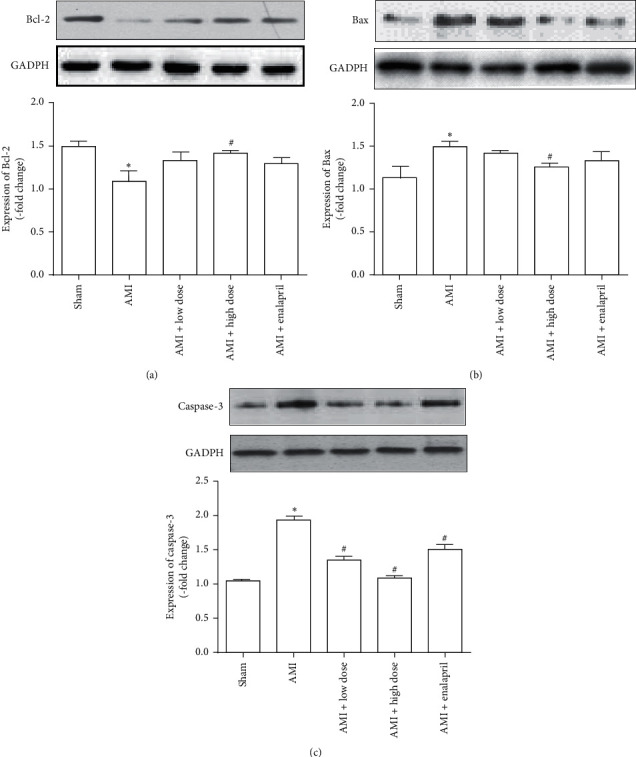
Effects of HQJZT on the expression of Bcl-2, Bax, and Caspase-3. (a) Expression of the antiapoptotic protein Bcl-2, upper is the representative blots of Bcl-2 and GAPDH; bottom is the densitometric analysis of Bcl-2 expression normalized to GAPDH (*n* = 6). (b) Expression of the proapoptotic protein Bax, upper is the representative blots of Bax and GAPDH; bottom is the densitometric analysis of Bax expression normalized to GAPDH (*n* = 6). (c) Expression of Caspase-3, upper is the representative blots of Caspase-3 and GAPDH; bottom is the densitometric analysis of Caspase-3 expression normalized to GAPDH (*n* = 6). ^*∗*^*P* < 0.05 compared with sham group. ^#^*P* < 0.05 compared with AMI group.

Bcl-2 and Bax genes are reported to play a crucial role in cell survival or death after apoptotic stimuli. Caspase-3 is also an important component of the apoptotic signaling pathway. The effects of HQJZT on Bcl-2, Bax, and Caspase-3 expression in myocardial tissues were analyzed by western blot. AMI significantly reduced the expression of Bcl-2 protein and increased the expressions of Bax and Caspase-3 proteins. Treatment with high dose of HQJZT increased the expression of Bcl-2 when compared with AMI group (Figure 4(a)) and decreased the expression of Bax when compared with AMI group (Figure 4(b)). Either low or high dose of HQJZT treatment reduced the expression of Caspase-3 when compared with AMI group (Figure 4(c)). In addition, our results showed that treatment with enalapril only changed the Caspase-3 expression, but not Bcl-2 and Bax expressions. These results indicate that HQJZT may play its therapeutic role via its antiapoptotic effect.

## 4. Discussion

In our present study, we clearly demonstrate that HQJZT improves cardiac function and reduces myocardial infarction injury. The related mechanism may be through regulation of cell apoptosis cascades (increase of Bcl-2 and decrease of Bax and Caspase-3 expression).

HQJZT used in the present study is composed of seven herbs: *Radix Astragali*, *Radix Paeoniae Alba*, *Ramulus Cinnamomi*, *Fructus Jujubae*, *Glycyrrhizae Radix Et Rhizoma Praeparata Cum Melle*, *Rhizoma Zingiberis Recens*, and *Saccharum Granorum.* Each has been widely used in traditional Chinese medicine for treatment of various diseases. *Radix Astragali* injection has been demonstrated to improve immune function in patients with congestive heart failure (CHF); it was proposed to be taken as an important auxiliary treatment for CHF [22]. Effects of *Radix Astragali* on patients with chronic viral hepatitis B [23], primary glomerulonephritis [24], and systemic lupus erythematosus [25] have been reported. *Radix Paeoniae Alba* has been used to treat coronary heart disease [26] and primary Sjögren's syndrome [27]. *Ramulus Cinnamomi* was applied for treatment with hysteromyoma [28], and it was proved to improve the efficacy of malignant pleural effusion treatment [29]. The effect of *Fructus Jujubae* fruits on dyslipidemia in obese adolescents has been investigated, showing potential favorable effects on serum lipid profile [30]. Antiepileptic effect of *Fructus Jujubae* was also observed [31]. Oil from *Glycyrrhizae Radix Et Rhizoma Praeparata Cum Melle* has effects with respect to increasing muscle mass and suppressing the percentage of body fat in elderly populations [32]. *Glycyrrhizae Radix Et Rhizoma Praeparata Cum Melle* has been used in the treatment of seasonal influenza [33] and Parkinson's disease [34]. Clinical trial has been conducted to assess the effects of *Rhizoma Zingiberis Recens* on depressed patients [35] and postinfectious cough patients [36]. *Saccharum Granorum* has been shown to improve the gastrointestinal tolerability of miglustat and reduce the magnitude of changes in body weight, particularly if initiated at or before the start of therapy [37]. The above-mentioned clinical data revealed that these herbs are not only widely used, but also effective and safe.

It is well known that myocardial infarction inevitably results in damage of heart tissue and reduction of heart function [38,39]. From this point of view, how to reduce heart tissue damage and recover heart function in myocardial infarction patients is essentially important. Our present investigation shows that cardiac function (±d*p*/d*t*_max_) was improved after one-week treatment with HQJZT (low dose or high dose, Table 2). In addition, our results also show that tissue injury was ameliorated after the treatment of HQJZT for one week both in low and high dose (Figure 2). It should be noted that low dosage of HQJZT treatment showed less effect on cardiac function, only improving −d*p*/d*t*_max_; even though it markedly reduced tissue injury, our present study at least proves that HQJZT improves cardiac performance in cardiac infarction model, which is maybe dose dependent.

Traditionally, it has been well recognized that cellular response to myocardial infarction is highly related to the activation of apoptotic signaling pathway [40]. Various strategies have been explored to suppress the activation of apoptosis [41,42]. Our present data shows that HQJZT reduces apoptotic cell number (Figure 3) and has significant effect on regulation of antiapoptotic protein Bcl-2, proapoptotic protein Bax, and Caspase-3 (Figure 4); these data imply that HQJZT may play its therapeutic role via regulation of apoptotic signaling pathway.

In the present study, we applied enalapril as a positive control; however, although enalapril treatment also improves the cardiac function, compared with the HQJZT, it shows less effectiveness. In addition, enalapril treatment does not show reduction in cardiac tissue injury. It should be noted that enalapril has been widely demonstrated to play a protective role in cardiac infarction model, although our present study shows less effectiveness compared with HQJZT; it may be due to the dose applied in the present study; this dose of Enalapril has been demonstrated to improve hemodynamic parameters in chronic left ventricular dysfunction rat model [21]. It also should be addressed that our present observation period is only one week, because it is crucially important to recover from infarction as soon as possible; long-time effects of HQJZT should be better and broader to evaluate its therapeutic role in response to cardiac infarction model, especially in mortality data, which shows no significant improvement in the present study.

## 5. Conclusion

In conclusion, our present observation demonstrates that HQJZT improves cardiac function and ameliorates cardiac injury, which may be through the regulation of apoptotic signaling pathway.

## Figures and Tables

**Table 1:** Composition of HQJZT.

Chinese name	Plant name	English name	Amount (g)	Place of origin
Huang Qi	*Astragalus membranaceus* (Fisch.) Bunge	Radix Astragali	30	Inner Mongolia, China
Bai Shao	*Paeonia lactiflora* Pall.	Radix Paeoniae Alba	15	Zhejiang, China
Gui Zhi	*Cinnamomum cassia* (L.) J. Presl	Ramulus Cinnamomi	15	Guangdong, China
Da Zao	*Ziziphus jujuba* Mill.	Fructus Jujubae	5	Guangdong, China
Zhi Gan Cao	*Glycyrrhiza uralensis* Fisch.	Glycyrrhizae Radix Et Rhizoma Praeparata Cum Melle	15	Jiangsu, China
Sheng Jian	*Zingiber officinale* Roscoe	Rhizoma Zingiberis Recens	10	Jiangsu, China
Yi Tang	*Saccharum Granorum*	Maltose	30	Guangdong, China

**Table 2:** Cardiac function parameters.

	Sham (*n* = 10)	AMI (*n* = 10)	AMI + low dose (*n* = 10)	AMI + high dose (*n* = 10)	AMI + enalapril (*n* = 10)
HR (bpm)	343.9 ± 160.5	337.0 ± 87.7	289.4 ± 92.8	340.156 ± 56.6	325.2 ± 122.1
RRI (ms)	298.1 ± 337.4	217.8 ± 35.5	236.4 ± 111	220.8 ± 13.2	201.6 ± 49.7
SAP (mmHg)	90.4 ± 9.1	61.9 ± 31.1	77.1 ± 24.9	91.5 ± 23.6	65.8 ± 13.5
DAP (mmHg)	84.1 ± 3.7	43.3 ± 29.1^*∗*^	73.0 ± 23.1	75.4 ± 10.8^#^	57.7 ± 5.4
MAP (mmHg)	87.7 ± 4.0	52.7 ± 31.1^*∗*^	76.6 ± 24.8	80.8 ± 17.8	67.2 ± 5.5
PP (mmHg)	6.5 ± 3.7	16.3 ± 6.3^*∗*^	4.1 ± 4.0	19.5 ± 17.5	6.5 ± 9.9
*P*_max_ (mmHg)	105.0 ± 11.6	66.8 ± 25.3^*∗*^	90.7 ± 17.6	94.6 ± 13.9	78.6 ± 14.1
*P*_min_ (mmHg)	10.5 ± 6.3	47.8 ± 22.9	69.7 ± 8.3	80.4 ± 20.8	82.1 ± 24.7
*P*_mean_ (mmHg)	54.9 ± 6.2	52.9 ± 25.2	76.4 ± 18.3	76.2 ± 18.9	67.8 ± 8.8
d*p*/d*t*_max_ (mmHg/s)	3825.3 ± 1067.7	1419.9 ± 488.1^*∗*^	1600.8 ± 208.4	2441.0 ± 186.7^#^	1600.8 ± 208.4
−d*p*/d*t*_max_ (mmHg/s)	−3711.1 ± 1225.2	−999.5 ± 248.2^*∗*^	−1504.8 ± 58.4^#^	−1402.8 ± 250.8^#^	−1502.0 ± 62.8^#^
LVEDP (mmHg)	69.8 ± 29.1	51.8 ± 25.6	76.4 ± 18.3	80.4 ± 23.3	78.2 ± 9.9

## Data Availability

The data used to support the findings of this study are available from the corresponding author upon request.

## Abbreviations

HQJZT: Jian Zhong Qiang Xin granules
AMI: Acute myocardial model
i.p.: Intraperitoneal injection
HR: Heart rate
RRI: The R-R interval
SAP: Systolic arterial pressure
DAP: Diastolic arterial pressure
MAP: Mean arterial pressure
PP: Pulse pressure
*P*_max_: The maximum of left ventricular pressure development
*P*_min_: The minimum of left ventricular pressure development
*P*_mean_: The mean of ventricular pressure development
LVEDP: Left ventricular end-diastolic pressure
+d*p*/d*t*_max_: Rates of maximum positive left ventricular pressure development
−d*p*/d*t*_max_: Rates of maximum negative left ventricular pressure development.
